# Liquorice Cultivation

**Published:** 1889-09

**Authors:** 


### LIQUORICE CULTIVATION.

Another branch of agriculture has been found possible in the climate and soil of California. It is the raising of the liquorice plant (glycyrrhiga.) It was tried last year in California with great success. The plant belongs to the natural order *leguminosae*, and is noted for its long, pliant, sweet roots from whence it derives its Greek name, *sweet root.* From these roots the liquorice of commerce is extracted.

Liquorice is propagated by slips like horse-radish. It requires a well fertilized soil, broken and mellow to the depth of three feet. The best roots are obtained by encouraging them to strike downward to a great depth. The plants are set in rows two feet apart and five or six inches apart in the row, and it takes three years for the roots to become ready for use, during which time the plant must be kept well fertilized and free from weeds. Digging the roots, which must be done with a spade, is a serious matter. The whole plant is taken up, the ground being thoroughly dug over to the depth of three feet: the main roots are selected for medical use, and the side roots used for futher propagation.

Above ground the plant throws up stems three and four feet high. Its leaves are pinnated, with many leaflets terminating in an odd one; flowers, whitish violet colored, in spikes, or racemes: a 5-cleft, 2-lipped calyx and a 2-leaved keel. When the growing season is over the old stalks are all removed and the ground covered with a rich compost.

Hitherto the plant has been cultivated principally in southern Europe, although to some extent in Asia and also in the southern part of England. From most of these places it comes in the form of the black extract. The United States, however, imports vast quantities of the root for extraction.

But a short time ago New York in one week received over 100 tons of the root from one place—Smyrna.

If California succeeds in supplying our home market with liquorice she has a large order to fill. Gray's botany reports an American species, *G. lepidota* growing wild in the sands of the shore near Buffalo N. Y.—*Teacher's Outlook.*

